# Lipid binding proteins from parasitic platyhelminthes

**DOI:** 10.3389/fphys.2012.00363

**Published:** 2012-09-12

**Authors:** Gabriela Alvite, Adriana Esteves

**Affiliations:** Faculty of Sciences, Biochemistry Section, Department of Cell and Molecular BiologyUdelaR, Montevideo, Uruguay

**Keywords:** platyhelminthes, FABPs, HLBPs, *Echinococcus*

## Abstract

Two main families of lipid binding proteins have been identified in parasitic Platyhelminthes: hydrophobic ligand binding proteins (HLBPs) and fatty acid binding proteins (FABPs). Members of the former family of proteins are specific to the Cestoda class, while FABPs are conserved across a wide range of animal species. Because Platyhelminthes are unable to synthesize their own lipids, these lipid-binding proteins are important molecules in these organisms. HLBPs are a high molecular mass complex of proteins and lipids. They are composed of subunits of low molecular mass proteins and a wide array of lipid molecules ranging from CoA esters to cholesterol. These proteins are excretory-secretory molecules and are key serological tools for diagnosis of diseases caused by cestodes. FABPs are mainly intracellular proteins of low molecular weight. They are also vaccine candidates. Despite that the knowledge of their function is scarce, the differences in their molecular organization, ligand preferences, intra/extracellular localization, evolution, and phylogenetic distribution, suggest that platyhelminths HLBPs and FABPs should play different functions. FABPs might be involved in the removal of fatty acids from the inner surface of the cell membrane and in their subsequent targeting to specific cellular destinations. In contrast, HLBPs might be involved in fatty acid uptake from the host environment.

## Introduction

Long chain fatty acids (LCFA) are involved in various cellular processes, including membrane synthesis, control of energy supply, and protein modification. LCFA and some of their active metabolites also function as signaling and regulatory molecules; they facilitate a dynamic interplay between the extracellular media, cellular membranes, cytoplasmic stores, and nuclei to control multiple biological activities. The hydrophobic nature of LCFA renders them poorly soluble in aqueous solution, so their intracellular transport to sites of metabolism and action is believed to be mediated by lipid binding proteins.

Parasitic helminths express high levels of lipid binding proteins and are incapable of *de novo* synthesis of fatty acids and cholesterol (Smyth and McManus, 1989 and references therein). They depend largely on the sequestration and utilization of host lipids during infection to survive. It is therefore essential that these parasites have an efficient binding system for the uptake and transport of key hydrophobic molecules. In this metabolic context, lipid-binding proteins might play an important role in the exchange of lipids between parasite and host organism. These proteins might also be involved in the uptake, transfer, and storage of hydrophobic ligands, in the targeting of ligands to specific organelles or pathways, in the sequestration of toxic compounds, and in the regulation of gene expression.

**Figure 1:**
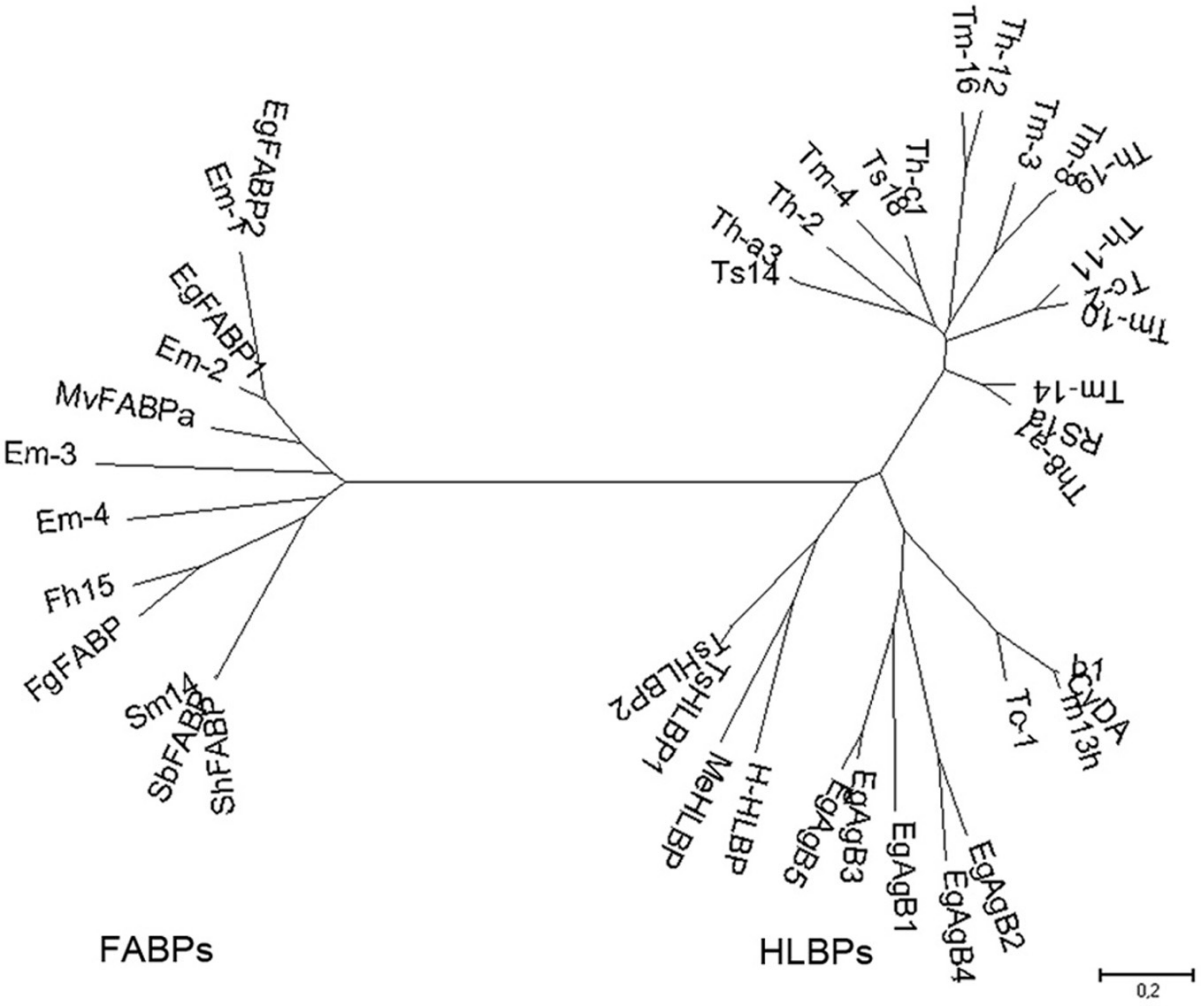
**Phylogenetic relationships among platyhelminth FABPs and HLBPs.** Consensus tree derived from neighbor joining analysis of the following sequences: EgFABP1 and EgFABP2 from *Echinococcus granulosus*; Em-1 (EmW_000549800), Em-2 (EmW_000550000), Em-3 (EmW_000551000), and Em-4 (EmW_000417200) from *E. multilocularis* (http://www.genedb.org/); Fh15 from *Fasciola hepatica*; FgFABP from *Fasciola gigantica*; Sm14 from *Schistosoma mansoni*; SbFABP from *Schistosoma bovis*; ShFABP from *Schistosoma haematobium*; MvFABPa from *Mesocestoides vogae*; EgAgB1-5 (AAD38373, AAS88249, ACO24475, AAS88245, AAW78441) from *E. granulosus*; HLBPs subunits RS1 (JF906191), Ts14 (JF906194), Ts18 (JF906196), CyDA (AEP03196.1), b1 (JF906188.1), and m13h (AAF06716.1) from *T. solium* larvae; TsHLBP1 (JF732995) and TsHLBP2 (JF32996) from adult *T. solium*; Tc1-2 (U07150, ACI42329) from *T. crassiceps*; H-HLBP (AF249884) from *H. diminuta*; MeHLBP (AF312736.1) from *M. expasa*; Tm-3 (ACI42324.1), Tm4 (ACI42322.1), Tm8 (ACI42321.1), Tm10 (ACI42329), Tm14 (ACI42323.1), and Tm16 (ACI42332.1) from *T. multiceps*; Th-a1 (ACI42323.1), Th-c1 (AAK21960.1), Th-a3 (ABI20731.1), Th11 (ACI42359), Th12 (ACI42360.1), Th19 (ACI42364.1), and Th2 (ACI42351.1) from *T. hydatigena.* GenBank accession numbers are indicated when many variants have been annotated.

Two groups of lipid binding proteins have been studied extensively in platyhelminth parasites: hydrophobic ligand binding proteins (HLBPs), and fatty acid binding proteins (FABPs). Both family members share the ability to bind lipids, but they differ in their ligand binding specificity, sequence, structure, and putative function. Phylogenetic studies indicate that HLBPs and FABPs evolved through different pathways and have discrete evolutionary origins (Figure 1). Members of both groups are putative targets for chemotherapy, vaccine development and immunodiagnostics.

## HLBPs

HLBPs form a family of cestode-specific lipoproteins. Two main classes of HLBPs have been described: one class consists of molecules that are confined to the cytoplasm, whereas the other class consists of molecules that are secreted and/or excreted. HLBPs were identified as highly abundant, immunogenic, and high molecular mass oligomers whose monomers were helix-rich subunits of approximately 7–11 kDa. It has been proposed that HLBPs might play an important role in the biological function of cestodes by controlling the sequestration of lipids from the host organism and also by regulating drug sequestration. In addition, HLBPs might act as messenger molecules. For example, HLBPs could bind to signaling lipids and subsequently participate in cell activation and/or differentiation processes that are required for parasite adaptation to host immune responses.

Members of the HLBP family of proteins have been identified in *Echinococcus granulosus* (EgAgB) (Oriol et al., 1971), *Taenia solium* (TsHLBPs) (Sako et al., 2000; Lee et al., 2007; Kim et al., 2011), *Moniezia expansa* (MeHLBP) (Jansen and Barrett, 1995; Barrett et al., 1997), *Himenolepys diminuta* (H-HLBP) (Saghir et al., 2000, 2001), and in *Taenia crassiceps* (Tc-HLBP) (Zarlenga et al., 1994). Genes sharing high sequence identity with other members of the HLBP family have been identified in *Taenia hidatigena* (ThLBPs) and *Taenia multiceps* (TmHLBPs); their sequences have been deposited in GenBank (Wan-zhong et al., 2011). HLBPs are capable of binding fatty acids and their CoA esters, triacylglycerols, sterols, lysophospholipids, phospholipids, and a range of non-polar organic ions and anthelmintic drugs. The diagnostic value of HLBPs has been studied extensively, whereas the biological function of these proteins has received less attention. Interestingly, there is some sequence similarity between ABC transporters, a *Granulicella tundricola* permease, and HLBPs (as determined by sequence comparison using DELTA-BLAST). The relationship between all members of the HLBP family is shown in Figure 1.

Antigen B was initially identified in hydatid cyst fluid derived from *E. granulosus* (EgAgB) (Oriol et al., 1971). It is highly immunogenic and is a major component of hydatid cyst fluid; it accounts for 90% of purified antigens. Moreover, EgAgB is one of the antigens that are currently used in the serodiagnosis of human cystic echinococcosis. It is a polymeric protein that has a molecular weight of 160 kDa and is composed of at least five 8 kDa subunits (EgAgB8/1–EgAgB8/5) (González et al., 1996; Chemale et al., 2001; Arend et al., 2004). The EgAgB8/2 subunit is the most effective for serodiagnosis (Rott et al., 2000; Virginio et al., 2003). Proteins homologous to these EgAgB subunits have been described in *Echinococcus multilocularis* (EmAgB8/1–EmAgB8/5) (Frosch et al., 1994; Mamuti et al., 2004, 2006). The *EmAgB8* genes are differentially transcribed during different developmental stages of the parasite (Mamuti et al., 2006).

Little information is available regarding the *in vivo* function of antigen B. However, *in vitro* experiments identified parasite protease inhibitor activity and immunoregulatory function of EgAgB, suggesting that EgAgB may modulate host defense by down-regulating neutrophils and dendritic cell-mediated innate responses, as well as T-cell-dependent responses (Shepherd et al., 1991; Rigano et al., 2001, 2007; Virginio et al., 2007).

Recently, Obal and co-workers analyzed the lipid composition of immunopurified bovine and human EgAgB. Interestingly, lipids from diverse lipid classes were identified; triacylglycerides, a variety of phospholipids (mainly phosphatidylcholine), and cholesterol were particularly abundant (Obal et al., 2012). EgAgB is predicted to adopt an organized structure than can accommodate lipid molecules within a single polymeric complex, similar to animal plasma lipoproteins. This might explain the heterogeneity in the molecular mass of molecules that can be accommodated by EgAgB (Chapman, 1980; Obal et al., 2012).

HLBP function has also been studied extensively in *T. solium*; HLBPs have been isolated from both larval and adult stages of this organism. HLBPs in *T. solium* are polymeric molecules consisting of protein subunits encoded by two gene families. One gene family encodes subunits with a molecular weight of 7 kDa (*RS1, Ts14, Ts18*), whereas the other gene family encodes 10 kDa subunits (*CyDA, b1, m13h, TsHLBP1, TsHLBP2*) (Kim et al., 2011; Rahman et al., 2012). These subunits preferentially bind different hydrophobic ligands, which is likely a reflection of their diverse primary structure and/or differential adaptation.

*T. solium* metacestode larvae express a 150 kDa hetero-oligomeric protein complex that is composed of multiple subunits of 7, 10, and 15 kDa, the latter representing a glycosylated form of the 10 kDa subunit. This protein complex appears to have an excretory-secretory function. Interestingly, each subunit has a different propensity to bind antibodies in the sera of infected patients. It has been proposed that this complex might be expressed and excreted exclusively in viable metacestodes. The hetero-oligomeric protein described above is capable of binding hydrophobic ligands and has also been shown to colocalize with lipid droplets and lipase activity at the host granuloma wall. The authors of the aforementioned study suggested that after fatty acid binding, the protein-lipid complex might return to the parasite across the syncytial membrane (Lee et al., 2007). Proteins belonging to the Ts14 and Ts18 sub-classes are known to multimerise to form an alternative 120 kDa complex, whose biological function has not yet been elucidated (Lee et al., 2005). The adult taenia express a protein complex of 100 kDa, whose major components are subunits of 10 kDa, coded by the genes *TsHLBP1* and *TsHLBP2*. This protein has been identified as an excretory-secretory product and appears to be produced exclusively in adults. Its binding properties are similar to those of known HLBPs.

*M. expansa* HLBP (MeHLBP) shares some similarity with antigen B proteins from *E. granulosus* and *E. multilocularis*, and with antigens expressed in *T. crassiceps*. Thirty-one subunits of 8 kDa each have been shown to aggregate in solution to produce a polymeric MeHLBP molecule of 250 kDa. Three-dimensional analysis using recombinant protein suggests that the 8 kDa subunit is composed of four helices. Binding experiments indicate that the recombinant subunit possesses a unique binding site that is able to bind a wide range of hydrophobic ligands, including long-chain fatty acids, sterols, retinoids, and anthelmintic (bithionol, hexachlorophene, niclosamide, nitroscanate, and oxyclozanide). Despite similarities in sequence with HLBPs expressed in other species, there is no evidence that MeHLBP is secreted *in vivo* or that it is glycosylated (Jansen and Barrett, 1995).

A highly abundant cytoplasmic HLBP (H-HLBP), of the same type as MeHLBP, was isolated from the tapeworm *Hymenolepis diminuta*. The purified protein produced a single band at 11 kDa when subjected to electrophoresis under reducing conditions. Binding assays indicated that H-HLBP was able to bind a wide range of hydrophobic ligands, including anthelmintic. Fluorescence studies suggested the existence of a single ligand-binding site (Saghir et al., 2000, 2001).

## FABPs

FABPs are another family of lipid binding proteins that have been the subject of study. These mainly intracellular proteins have a highly immunogenic character that serves to confer significant levels of protection against challenge infections, and has established FABPs as vaccine candidates (Esteves, 2009 and references herein). These proteins, unlike HLBPs, are widely distributed across animal species. Although the function of FABPs has not been fully elucidated, the results of experimental work conducted in vertebrates have contributed to our understanding of the role of these proteins in parasites. Currently, there is no evidence to support the presence of FABPs in the sera of infected patients.

The first platyhelminth FABP to be described was isolated from the parasite *Schistosoma mansoni* and was designated Sm14 due to its apparent molecular mass (Moser et al., 1991). Homologous proteins from *Schistosoma japonicum* (Sj-FABPc) (Becker et al., 1994), *Schistosoma bovis* (SbFABP) (GenBank Accession Number: AY6 15730), *Fasciola hepatica* (Fh15) (Rodríguez-Pérez et al., 1992), *Fasciola gigantica* (FgFABP) (Estuningsih et al., 1997), *E. granulosus* (EgFABP1 and EgFABP2) (Esteves et al., 1993, 2003), *Mesocestoides vogae* (MvFABPa and MvFABPb) (Alvite et al., 2008), and *T. solium* (TsFABP) (GenBank Accession Number ABB76135), were subsequently isolated and characterized.

Platyhelminth FABPs share low amino acid sequence identity with vertebrate FABPs; in addition, they do not contain extensive common protein sequence motifs (Esteves et al., 1997). However, solved or predicted 3D structures for platyhelminth FABPs are similar to the typical β-barrel structure previously resolved for their vertebrate FABP counterparts (Jakobsson et al., 2003; Angelucci et al., 2004). Interestingly, rEgFABP1 and rSm14 accommodate fatty acid (FA) ligands in the U-shaped conformation that has been observed for heart type FABPs (H-FABPs) (Jakobsson et al., 2003; Angelucci et al., 2004). The vertebrate FABP residues that have been implicated in binding of the carboxylate group of fatty acid ligands (Arg 106, Arg 127, Tyr 129) are conserved in platyhelminth FABPs, with the exception of FABPs expressed in *Fasciola*.

Platyhelminth FABP protein sequences appear to share greater similarity to those belonging to the large group of vertebrate FABPs (consisting of CRABPs/CRBPs/H-FABP/B-FABP/E-FABP/TLBP/ALBP/MLBP) compared to those isolated from intestine and liver combined (Esteves et al., 1997).

Liver type FABPs (L-FABPs) and intestine-type FABPs (I-FABPs) appear to have originated approximately 930 million of years ago as a result of the duplication of the primordial iLBP gene. Around the time of divergence of vertebrate and invertebrate lineages (700–600 mya), CRABPs and H-FABPs lineages are presumed to have already been in existence (Schaap et al., 2002). Interestingly, the FABPs characterized in platyhelminths, which are the most distant ancestral FABPs known to date, appear to share the highest identity with vertebrate CRABPs and HFABPs (Esteves et al., 1997). The limited number of ancestral FABPs suggests that these molecules should have a low specificity for ligands and a large repertoire of possible binding partners within the cell. In this context, it is likely that the small group of ancestral FABPs might be capable of satisfying all of the functions fulfilled by the larger family of vertebrate FABPs. Functional specialization might have resulted from subtle changes produced in the internal cavity or at the surface, thereby favoring the interaction of FABPs with specific ligands (Esteves and Ehrlich, 2006). Alternatively, the possibility that platyhelminth genomes contain genes encoding liver and intestine type FABPs cannot be excluded. Currently, there is no complete sequence data available to conclusively support or exclude the expression of these genes in platyhelminths; however, neither liver nor intestine type FABP genes have been identified in the almost complete *E. multilocularis* genome (http://www.sanger.ac.uk/cgi-bin/blast/submitblast/Echinococcus).

The traditional view of FA transport across biological membranes is that it occurs by passive diffusion. Evidence is accumulating, however, that several membrane-associated proteins, including mammalian FAT/CD36, FABPpm and FAT, are involved in membrane transport of FAs (van der Vusse et al., 2002). These proteins have not yet been reported in Platyhelminthes. However, mining of *E. multilocularis* sequence using the *E. multilocularis* BLAST server (http://www.sanger.ac.uk/cgi-bin/blast/submitblast/Echinococcus) identified sequences similar to all three membrane-associated FA transport proteins (CD36/FAT, FATP and FABPpm). A sequence of *S. japonicum* annotated in GenBank, baring high sequence identity with CD36/FAT, was also identified in *E. multilocularis* (Alvite and Esteves, 2011).

Vertebrate FABPs are predicted to play an important role in facilitating the incorporation and intracellular transport of fatty acids. They are promising candidates for FA removal from the inner surface of the cell membrane and for FA transfer to the appropriate intracellular compartment. Two distinct mechanisms for the transfer of free FAs from FABPs to artificial membranes have been proposed: the “collisional” mechanism (Kim and Storch, 1992a) and the “diffusional” mechanism (Kim and Storch, 1992b). The hypothesis that platyhelminth FABPs function in a similar manner to those in vertebrates was used to direct the study of the ligand transfer mechanism of platyhelminth FABPs. Based on this hypothesis, it was determined that FABPs from *S. japonicum* (McDermott et al., 2002), *E. granulosus* (Pórfido et al., submitted), and *M. vogae* (unpublished) transfer FAs via a collisional mechanism. The direct interaction of SjFABPc and EgFABP1 with artificial membranes was also demonstrated using a cytochrome c competition assay (McDermott et al., 2002; Pórfido et al., submitted).

**Figure 2:**
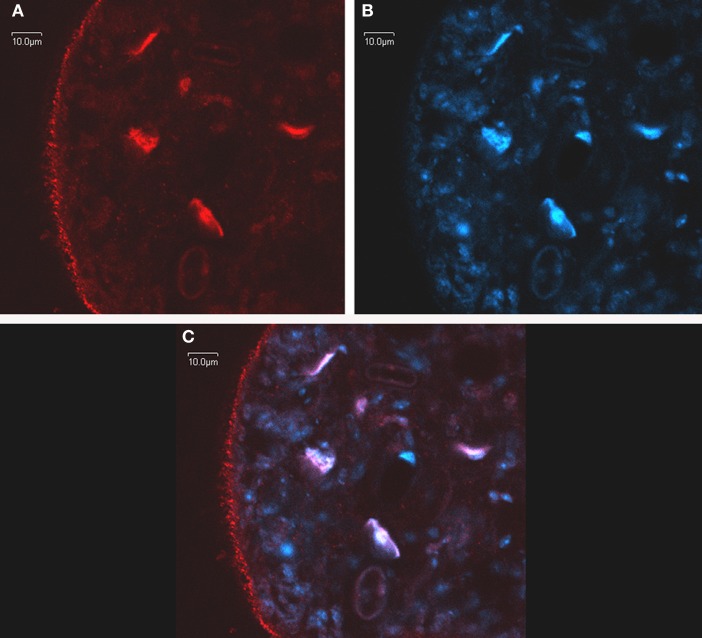
***In toto* confocal immunomicroscopy of *Mesocestoides vogae* larvae.** MvFABPs were visualized with an antibody against rEgFABP1 that recognizes both MvFABPs **(A)**. Nuclei stained with TO-PRO-3 (*Invitrogen*) **(B)**. Merged images from **(A)** and **(B)** showing colocalisation of MvFABPs and the nucleus. Violet regions indicate areas of colocalisation **(C)**. Images were taken at 90X magnification. Bars indicate 10 microns.

Immunomicroscopy studies from our group have demonstrated *in vivo* FA uptake in *M. vogae* tetrathyridia and have shown colocalization of MvFABPa and MvFABPb with the fluorescent fatty acid analog BODIPY FL-C_16_ (Molecular Probes, *Invitrogen*, Eugene, OR, USA). The widespread cytoplasmic distribution of MvFABPs and their colocalization with the fluorescent ligand indicate that these proteins are likely involved in ligand uptake, solubilisation and transport. Additional studies using intracellular markers demonstrated that MvFABPs participate in FA targeting to the mitochondria, endoplasmic reticulum/Golgi and nucleus (Figure 2). The observation that MvFABPs are involved in FA targeting to the mitochondria is intriguing because FAs are not employed for mitochondrial energy production. The colocalization of MvFABPa with the Golgi suggests a possible relationship with lipid transport and membrane synthesis.

The localization of MvFABPs and EgFABPs inside the nucleus indicates that these proteins might participate in the control of lipid metabolism by regulating the expression of genes involved in this metabolic process. Functional cooperation between FABPs and peroxisome proliferator activated receptors (PPARs), which are nuclear receptors, has been shown to regulate target gene transcription. PPARs control the expression of multiple genes involved in lipid and sugar metabolism, as well as genes that influence cell growth and differentiation. Hence, PPARs and their cognate FABPs mediate multiple cellular responses induced by their ligands. Substantial evidence shows the nuclear localization of some vertebrate FABPs, as well the direct interaction of FABPs with PPARs (Schroeder et al., 2008; Hostetler et al., 2009). Bioinformatic analysis using available sequences has also revealed the presence of the DNA-binding domain of the PPAR-like nuclear receptor family in the *Echinococcus* genome. In addition, a peroxisome proliferator activated response element (PRE) has been identified in the promoter region of EgFABP2 (Esteves et al., 2003).

Similar to vertebrate FABPs, no consensus nuclear localization signal has been identified in platyhelminth FABPs. However, conformational changes induced by ligand binding might generate a localization signal by modifying residues exposed at the surface of the protein (Hertzel and Bernlohr, 2000; Hostetler et al., 2005; Storch and Thumser, 2010). Ligands have been identified that appear to specifically induce nuclear translocation of adipocyte FABP (FABP4) (Gillilan et al., 2007). The Phe 57 side chain of FABP4 is proposed to adopt an open or closed conformation, exposing a nuclear localization signal comprised of Lys 22, Arg 30, and Lys 31. *In silico* analysis of EgFABP1 docked with FA showed that the Phe 58 side chain might adopt the open and closed conformations mentioned above and that linoleic, arachidonic, and oleic acid could function as activating ligands (Esteves and Paulino, 2012). Experimental work is in progress to evaluate whether any of these FAs can elicit a FABP-dependent biological response. Displacement assays indicate a preference for arachidonic acid and oleic acid over other fatty acids, phospholipids, and other hydrophobic ligands tested (Alvite et al., 2001).

## Concluding remarks

Platyhelminth HLBPs and FABPs are both involved in the sequestration and transport of lipids from the parasite host to the parasite. HLBPs are accumulated in cyst fluid and are also secreted into host tissue to participate in the uptake of a wide range of hydrophobic ligands; in contrast, FABPs are mainly intracellular proteins that preferentially bind fatty acids (Alvite et al., 2001; Storch and Thumser, 2010). HLBPs and FABPs differ in their molecular organization and do not share a close evolutionary relationship. HLBPs are specific to the Cestoda class of Platyhelminthes, while FABPs are found across animal species.

Studies of the biological function of HLBPs and FABPs are scarce. However, the diversity that exists among both LBP family members suggests that their functions are not redundant. HLBPs might translocate between parasite parenchyma cells (or cyst fluid) and host cells to transport free FAs, phospholipids, triacylglycerides, and cholesterol. In contrast to HLBPs, FABPs are proposed to participate in intracellular FA binding and transport. In concert with HLBPs, which in this context would sequester host FAs into the parasite, FABPs are proposed to remove FAs from the cell membrane inner surface and target them to specific cellular compartments. Taken together, the current evidence suggests that HLBPs and FABPs have distinct biological functions.

### Conflict of interest statement

The authors declare that the research was conducted in the absence of any commercial or financial relationships that could be construed as a potential conflict of interest.
